# Comparison of the contributions of the nuclear and cytoplasmic compartments to global gene expression in human cells

**DOI:** 10.1186/1471-2164-8-340

**Published:** 2007-09-25

**Authors:** Roger A Barthelson, Georgina M Lambert, Cheryl Vanier, Ronald M Lynch, David W Galbraith

**Affiliations:** 1 Bio5 Institute for Collaborative Bioresearch and Department of Plant Sciences, University of Arizona, Tucson, Arizona 85721, USA; 2 Department of Biological Sciences, University of Nevada, Las Vegas, Nevada 89154, USA; 3 Department of Physiology, University of Arizona, Tucson, Arizona 85721, USA

## Abstract

**Background:**

In the most general sense, studies involving global analysis of gene expression aim to provide a comprehensive catalog of the components involved in the production of recognizable cellular phenotypes. These studies are often limited by the available technologies. One technology, based on microarrays, categorizes gene expression in terms of the abundance of RNA transcripts, and typically employs RNA prepared from whole cells, where cytoplasmic RNA predominates.

**Results:**

Using microarrays comprising oligonucleotide probes that represent either protein-coding transcripts or microRNAs (miRNA), we have studied global transcript accumulation patterns for the HepG2 (human hepatoma) cell line. Through subdividing the total pool of RNA transcripts into samples from nuclei, the cytoplasm, and whole cells, we determined the degree of correlation of these patterns across these different subcellular locations. The transcript and miRNA abundance patterns for the three RNA fractions were largely similar, but with some exceptions: nuclear RNA samples were enriched with respect to the cytoplasm in transcripts encoding proteins associated with specific nuclear functions, such as the cell cycle, mitosis, and transcription. The cytoplasmic RNA fraction also was enriched, when compared to the nucleus, in transcripts for proteins related to specific nuclear functions, including the cell cycle, DNA replication, and DNA repair. Some transcripts related to the ubiquitin cycle, and transcripts for various membrane proteins were sorted into either the nuclear or cytoplasmic fractions.

**Conclusion:**

Enrichment or compartmentalization of cell cycle and ubiquitin cycle transcripts within the nucleus may be related to the regulation of their expression, by preventing their translation to proteins. In this way, these cellular functions may be tightly controlled by regulating the release of mRNA from the nucleus and thereby the expression of key rate limiting steps in these pathways. Many miRNA precursors were also enriched in the nuclear samples, with significantly fewer being enriched in the cytoplasm. Studies of mRNA localization will help to clarify the roles RNA processing and transport play in the regulation of cellular function.

## Background

Studies of global gene expression form an important component of a systems approach to understanding cellular function in normal and disease states. Although large-scale gene expression data serve to define the state of cellular systems [1], the perspective provided by any study of this type is necessarily limited by the experimental methods employed for measuring gene expression. For example, the transcriptome, defined as the entirety of all forms of RNA transcribed from the genome, can be conceptually and empirically subdivided into multiple parts, according to subcellular location. The methods used for studying the transcriptome can influence which subcellular compartments are included in subsequent analyses, and further, can determine what types of transcripts are included in the studies.

RNA is transcribed first within the nucleus, wherein it is accumulated to a steady state; this steady state is evidently a complex function of the rates of synthesis, processing, degradation, and export to the cytoplasm of the individual mRNAs [2,3]. Within the cytoplasm, the individual mRNAs accumulate to different steady state levels, according to their rates of export and to their different fates, including translocation to specific subcellular locations [4], translation on polyribosomes [3], sequestration within localized organelles such as P bodies [5,6] for storage and/or degradation mediated by microRNA (miRNA) and short-interfering RNA (siRNA) [7]. Conceptually, the levels of cytoplasmic RNAs, being located in the same compartment as the translational machinery, might be expected to correlate best with protein expression levels for proteins encoded within the nuclear genome. The transcript levels within the nuclear compartment, on the other hand, since they comprise newly-transcribed RNA albeit at much lower total amounts than the cytoplasm, might be expected to track most proximally the actively-transcribed portion of the chromatin, and therefore provide information concerning the most current transcriptional program for the cell. Empirically, nevertheless, global studies of gene expression, with few exceptions, employ RNA samples that are whole-cell extracts, and therefore are heavily weighted toward the contribution provided by cytoplasmic RNA.

Recent studies have illustrated a number of pitfalls associated with using only one cellular RNA source for transcriptome analysis. Cheng et al. [8] used Affymetrix tiling arrays to study both nuclear and cytoplasmic transcripts. They found that cytoplasmic RNA and nuclear RNA contained different, yet overlapping, populations of transcripts. Many of these transcripts represented portions of the genome that were not previously recognized as being, or predicted to be, transcribed, and included numerous transcripts in antisense orientations. Further, many of the transcripts in both pools were found to lack polyA sequences, which would preemptively remove them from any studies that use the polyA sequence to identify mRNA. This study by Cheng et al. and similar ones [9-13], coupled to the emerging importance of the regulatory activities of miRNA and siRNAs have considerably expanded our view of the transcriptome and of how it might function within the cell. For example, in the Cheng studies, 31.8% of all RNA transcripts were from unannotated, intergenic sequences, and 26% were intronic sequences. They found that nuclear RNA is especially rich in non-coding sequences, with 41% consisting of intergenic sequences and 25% intronic sequences. They also [8] determined that 41.7% of cellular transcripts were found only in the nucleus. Many of these transcripts were intronic or intergenic, polyA^- ^sequences; others included small nucleolar RNAs, alternative splicing forms, and primary transcripts for miRNA (pri-miRNA). Pri-miRNAs have been shown to reside almost entirely in the nucleus, where they initially are processed by the RNAse Drosha [14-16] prior to being exported into the cytoplasm in the form of double-stranded RNA (pre-miRNA). In the cytoplasm, pre-miRNA is processed further by the Dicer RNAse into small, single-stranded, mature miRNAs [14,17].

As we revise our view of the transcriptome, comparisons between nuclear and cytoplasmic RNA clearly serve to expand our understanding of the expression and regulation of even the best-annotated genes. Our pursuit of the following experiments was generally motivated by the practical goal of evaluating the validity of using isolated nuclei as a source of transcripts for gene expression studies, but was also coupled to an interest in a more-detailed understanding of the transcriptome. The interest in nuclear RNA as a source of transcriptional information stems from the empirical difficulties encountered in performing global studies of gene expression in important mammalian cell types that are interspersed within complex tissues. Existing experimental strategies to isolate interesting cells in this category, such as the beta cells within the Islets of Langerhans, which comprise only 5% of pancreatic cells, require dissociation from the matrix tissue by digestion with proteolytic enzymes, and invoke some method of cell separation and purification specific to the beta cells. The time and conditions required for processing the cells from tissue may severely compromise their gene expression programs. We considered that these problems might be mitigated if isolated nuclei, rather than separated cells, were used for cell-type specific gene expression studies, since nuclei can be isolated relatively rapidly from tissue under conditions where new transcription is halted. The basic approach is to tag nuclei in unique cell types with a fluorescent marker by the introduction of a Fluorescent Protein (FP) expressing transgene, driven by a cell type-specific promoter [18]. We and others have established that the Green Fluorescent Protein (GFP) can be efficiently targeted to the nucleus by fusion to topogenic sequences [19-23]. Intact nuclei then can be separated by homogenization at 4°C, and fluorescence activated sorting (FAS) [24,25]. Previous studies in plants have demonstrated the validity of this approach [22].

To explore the suitability of using nuclear RNA for global gene expression studies, we have compared global gene expression patterns derived from transcripts produced from nuclear and cytoplasmic extracts of the HepG2 human hepatoma cell line. Further, we have compared global gene expression patterns between transcripts from nuclear and total cellular extracts. We used human genomic microarrays for these studies, which provide a broad survey of the annotated portions of the transcriptome. Since many of the uniquely nuclear forms of RNA are not well represented on microarrays designed for gene expression, we also employed microarrays designed for analysis of miRNA expression [26]. We used them in a manner different from their designed purpose, by employing methods for transcript purification and amplification that exclude mature miRNAs, but that include the larger primary transcripts for miRNAs containing intact polyA sequences.

Our results indicate that global gene expression patterns based on microarray analyses are largely congruent for total, cytoplasmic, and nuclear RNA samples extracted from HepG2 cells. However, there were some significant differences between nuclear and cytoplasmic RNA; for this comparison, the reported transcript concentrations differed significantly between the compartments for 3% of the transcripts represented on the microarrays. Analysis of the annotation of transcripts that were significantly different between the nuclear and cytoplasmic fractions suggests they may play important roles in the control of key processes within the cell. A further finding from these experiments, that pri-miRNA transcripts were largely concentrated in the nucleus, is consistent with previous findings that pri-miRNA transcripts are processed in the nucleus prior to transport into the cytoplasm.

## Results

The HepG2 human hepatoma cell line was selected as a model system for transcript profiling within nuclear, cytoplasmic, and total RNA fractions. RNA fractions were prepared from four different passages of cells that were approximately 80% confluent, to provide some biological variability. Following amplification, labeled RNAs were hybridized to human genomic microarrays comprising 70-mer sense-strand array elements. The same RNA samples were also processed for hybridization using miRNA-specific microarrays.

**Figure 1:**
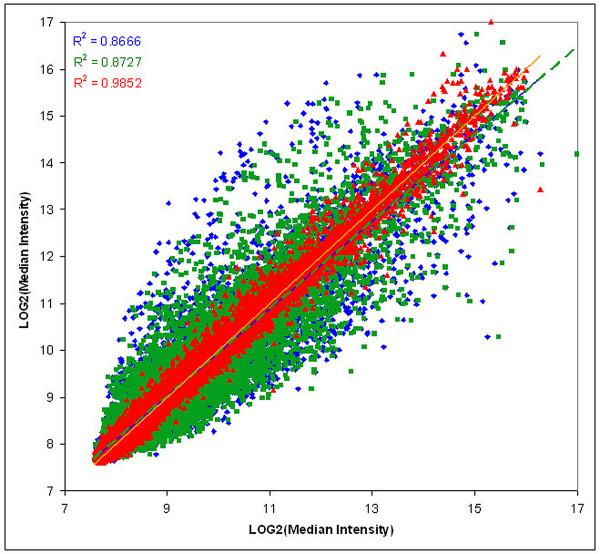
**Human genomic microarrays: a comparison of intensity values for nuclear, cytoplasmic, and total RNA samples**. The median intensity values from the hybridization of amplified RNA samples to the 70-mer probes on the human genomic microarrays were log-transformed and normalized. The least-squares mean log values from the mixed model ANOVA were plotted against each other to view the relative intensities for the following samples: Blue, nuclear (ordinate) versus cytoplasmic (abscissa) RNA; Green, nuclear (ordinate) versus total (abscissa) RNA; and Red, total (ordinate) versus cytoplasmic (abscissa) RNA. A least squares regression line was fitted to each set of points to visually demonstrate the linear relationship; the associated correlation coefficients are presented in colors that match the lines and the data points.

**Figure 2:**
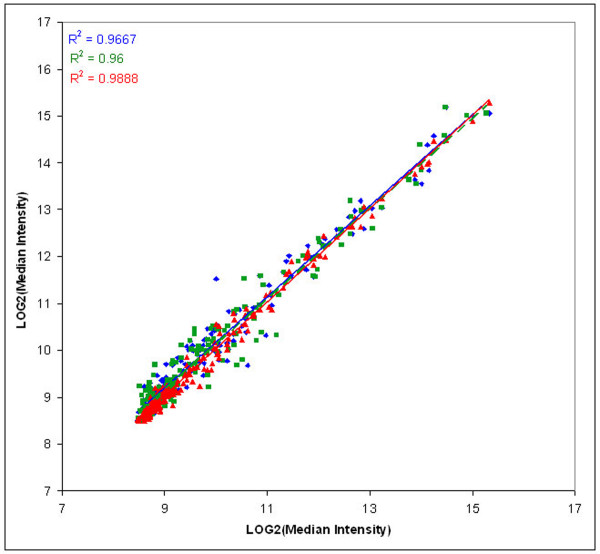
**MicroRNA microarrays: a comparison of intensity values for nuclear, cytoplasmic, and total RNA samples**. Sense DNA (reverse-transcribed, amplified RNA) samples were hybridized to the oligo probes (doublets of 18–22 nucleotides) on the MirMax miRNA microarrays. Hybridization of these samples to the miRNA arrays indicates the concentration of pri-miRNA in the RNA samples. The resulting intensity values were analyzed as given in Figure 1, and plotted with the same color key. A least squares regression line was fitted to each set of points to visually demonstrate the linear relationship; the associated correlation coefficients are presented in colors that match the lines and the data points.

Correlation plots of log median intensity values for nuclear, cytoplasmic, and total RNA were compared for both the human genomic and miRNA arrays (Figures 1 and 2). The high correlation coefficients imply a high degree of technical reproducibility of the overall microarray platform, including the amplification step, and a lack of biological variation across different samples. The greatest differences in transcript and miRNA levels were observed within comparisons of nuclear and cytoplasmic fractions. Given that a majority of the total cell RNA fraction comprises cytoplasmic RNA, this is not surprising. Overall, smaller magnitude differences were seen within the comparisons using the miRNA microarrays as compared to the genomic expression microarrays.

### Comparison of mRNA Isolated from Cytoplasmic and Nuclear Compartments

For the transcripts represented on the human genomic arrays, we tabulated those that displayed consistent differential expression between the nuclear and cytoplasmic fractions, as determined by analysis of variance (ANOVA). The criterion for significance was defined as a false discovery rate (FDR) less than or equal to 0.05 [see Methods and Additional file 1]. The transcripts meeting this criterion, a total of 743 (3.5%) out of the 21,383 represented on the array, were divided into two classes, those expressed at higher levels in the nucleus (389 transcripts), and those expressed at higher levels in the cytoplasm (354 transcripts). The magnitude of the enrichment of transcripts in the nuclear RNA fraction relative to the cytoplasm ranged from 1.14 fold to more than 12 fold, with 321 transcripts being more than 1.5-fold higher, and 192 more than 2-fold. For transcripts enriched in the cytoplasm relative to the nucleus, the range was from 1.16 to 5-fold, with 301 transcripts being more than 1.5-fold higher than in the nucleus, and 171 more than 2-fold.

**Figure 3:**
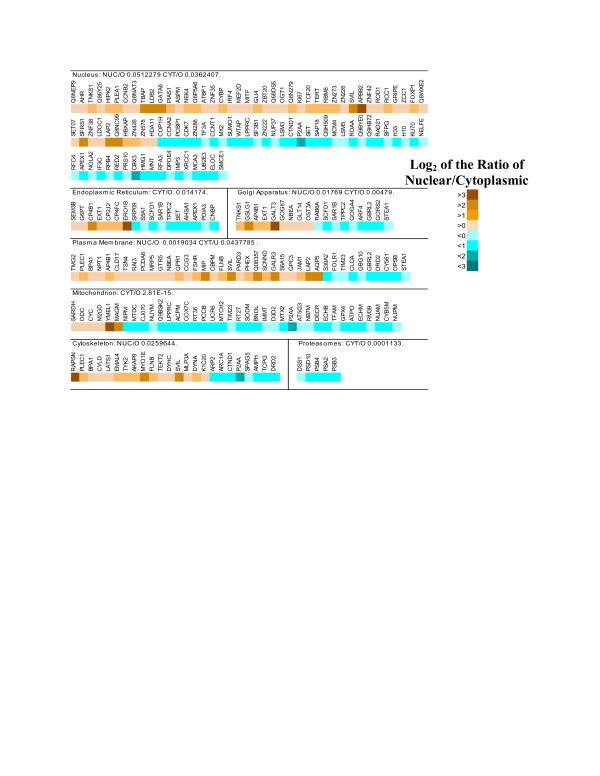
**GO cell component analysis of nucleus-enriched and cytoplasm-enriched transcripts**. The lists of nucleus-enriched (relative to cytoplasm) and cytoplasm-enriched (relative to nucleus) transcripts were calculated for the human genomic microarray data by ANOVA and by selection of those with a FDR less than 0.05. These two lists were submitted independently for analysis by GOToolbox [27] to determine the cell component annotation of the transcripts, and to determine whether some of the annotation categories were overrepresented on the lists, using the hypergeometric test with Benjamini and Hochberg FDR calculation. Some of the categories with strong representation among the transcripts are presented here. The transcripts that were placed in each category are identified by gene name or abbreviated TREMBL identifier and color-coded to indicate the ratio of the log, mean, normalized intensity values of the nuclear sample over the cytoplasmic sample. Where the lists for nuclear or cytoplasmic transcripts show overrepresentation in a GO category, the FDR is provided.

**Figure 4:**
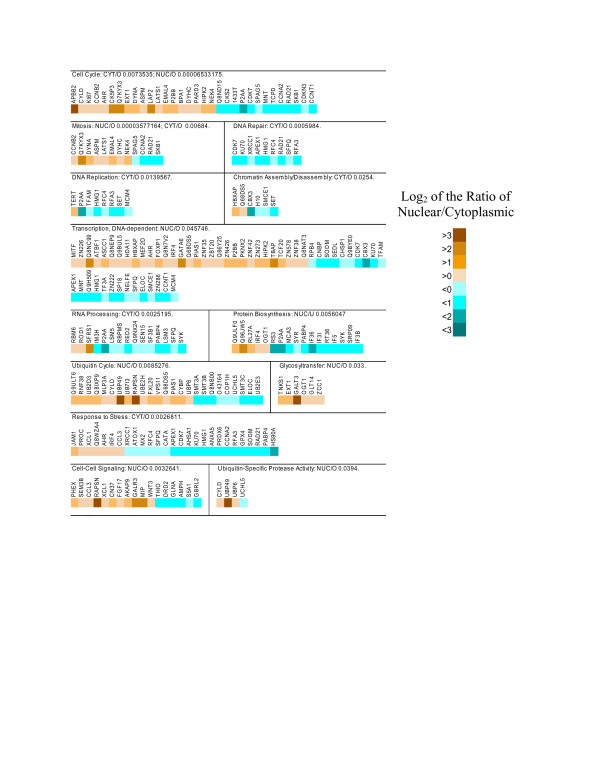
**GO biological process analysis of nucleus-enriched and cytoplasm-enriched transcripts**. Details are the same as for Figure 3, except the lists of transcripts were annotated using the biological process categories.

After annotation of the transcripts that were enriched in the nucleus, the gene ontology distributions were analyzed using the GOToolBox [27]. We searched for annotation classes that were overrepresented when compared to the human genome, as determined with a hypergeometric test, using the Benjamini and Hochberg correction to compensate for multiple testing [28,29]. Several annotation classes were overrepresented, including the GO cell component term nucleus (Figure 3). Many of the biological processes that were overrepresented were associated with the nucleus (Figure 4), including the cell cycle, mitosis, and transcription. Other classes overrepresented for transcripts enriched in the nucleus were for membrane-associated proteins, particularly those integral to the plasma membrane and the Golgi apparatus. Glycosyltransferases, which are generally membrane-associated proteins, also were overrepresented in the nuclear-enriched transcript fraction. Some GO headings related to the nucleus were represented but not significantly overrepresented among the nuclear-enriched transcripts, including those encoding chromatin assembly factors, and RNA processing enzymes. Finally, the class of transcripts associated with the ubiquitin cycle, discussed in more detail below, was also overrepresented in the list of nuclear-enriched transcripts.

When the transcripts that were enriched in the cytoplasm in comparison to the nucleus were analyzed, many of the same annotation classes found for nuclear-enriched transcripts were determined to be overrepresented in comparison to the whole genome, including the cell component 'nucleus' heading. Some of the overrepresented GO biological process classes for the cytoplasm-enriched transcripts included cell cycle, mitosis, metabolism, DNA repair, DNA replication, chromatin assembly/disassembly, and RNA processing. The cytoplasm-enriched transcripts also had overrepresentation in the cell component classes of membrane-associated genes, including endoplasmic reticulum, plasma membrane, and Golgi apparatus, as well as the classes mitochondrion, proteasomes, and response to stress. Other classes that were conspicuously represented but not enriched included the ubiquitin cycle, and transcription.

### Micro (mi)RNA Analysis

The same amplified RNA samples used with the human genomic microarrays were reverse-transcribed and labeled, so that they could be used with the MirMax miRNA microarrays, which consist of antisense, single stranded oligonucleotides representing 759 different miRNAs [26]. In these experiments, the RNA forms that were amplified [30] necessarily have a polyA tail, and are large enough to be purified by the Qiagen RNeasy procedure, which enriches for RNA greater than 200 nt in length. Mature miRNA and pre-miRNA, typically 22 nt and 70 nt in length respectively, are not purified or amplified efficiently under these conditions, but amplification of miRNA primary transcripts does occur, as they are polyadenylated, and are typically several hundred to several thousand nucleotides in length [16,31,32].

The fluorescence intensity data from scanning the MirMax arrays indicate that pri-miRNA is detected in these samples. Many of the human pri-miRNAs detected are for miRNAs that have been previously identified in liver cells, such as let-7b, miR-16, miR-92, miR-93, miR-122a, miR-125a, miR-125b, miR-150, miR-151, and miR-345 [33-35]. Human miR-122 has not been found in HepG2 cells previously, but our experiments do indicate that the primary transcript for miR-122 is present in our cultures [35].

Analysis of the miRNA hybridization data by ANOVA indicated differential miRNA accumulation between the nuclear and cytoplasmic RNA samples, at a FDR of less than 0.05, for 156 of the miRNA precursors. The Mirmax arrays are divided into subarrays that comprise probe sets for five species: human, mouse, rat, *D. melanogaster*, and *C. elegans*. Of the probes that showed significant differences, 116 were for non-human miRNAs. Of these, 36 were exact duplicates of probes for known human miRNAs, reflecting the high degree of cross-species conservation of some miRNAs [36,37]. In these cases, the probes for other species were technical replicates for the human miRNA probes, and the corresponding data reflected this. For example, hsa-miR-let7e had the highest nuclear to cytoplasmic ratio for human miRNAs, at 2.84, and the mouse and rat duplicates had ratios of 2.67 and 1.88, respectively.

Some of the probes for other species may identify novel human miRNAs; for example, the mouse probe for miR-207 is in the group showing significant differences, but no human homologue for this microRNA has been identified. The sequence for mouse miR-207 is found in the human genome. In other cases, such as for mouse miR-151 or Drosophila miR-34, the probes are different from those used for the human homologues, but these miRNAs have human counterparts [38,39]. For miR-151, the data for the human probes were very similar to the corresponding data for the mouse probes, but did not show significant differences in amounts between the nuclear and cytoplasmic RNA samples. For human miR-34, the data did show significant differences. For miR-34 and miR-151, the probes for the non-human microRNAs may be hybridizing to the human pri-miRNAs, but because the probe sequences are not designed specifically for the human homologues, the hybridized targets could be different microRNAs or other RNA sequences. Our samples hybridized strongly with the probes for both mouse and rat probes for miR-290, miR-292-5p, miR-297, miR-298, and miR-329. Only mouse miR-329 and rat miR-329 have a known human homologue, but there were no probes for the human miR-329 on this array. The human miR-329 was discovered in a search for sequences homologous to the rat miR-329 [40], but in our experiments, because the sequence is not identical to the sequence for the mouse or rat homologues, the rat and mouse probes may not have hybridized to a miR-329 primary transcript. Thus, each of the miRNA precursors hybridized by mouse or rat miRNA probes could potentially represent human homologues, but each must be examined on an individual basis to determine whether such homologues exist.

**Figure 5:**
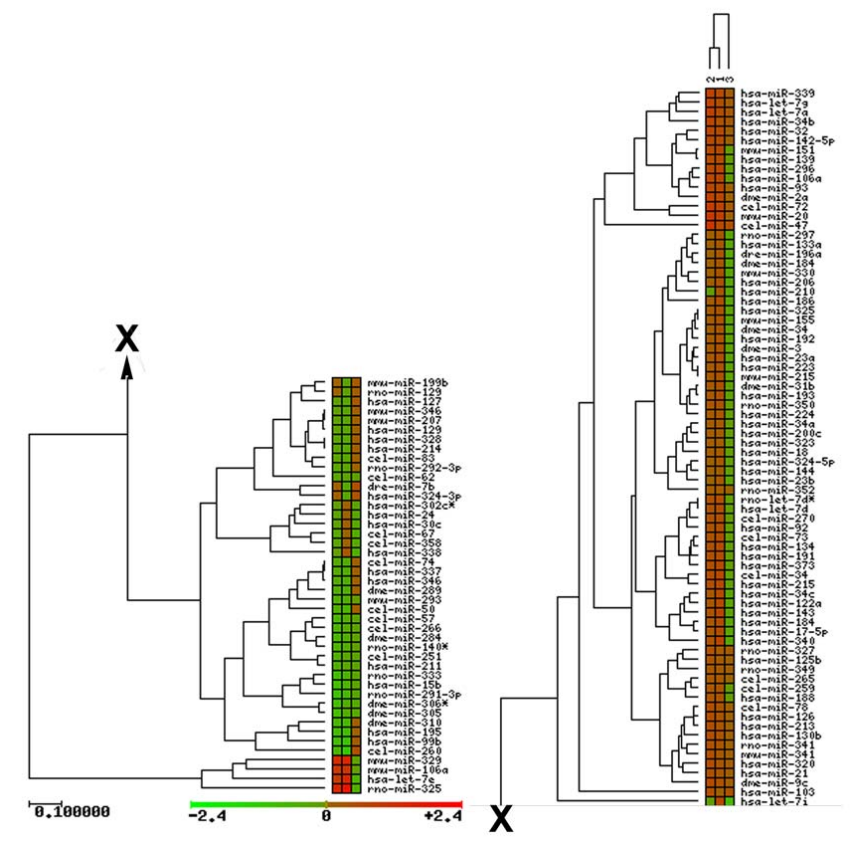
Cluster analysis of the miRNA primary transcripts identified in the nuclear, total, and cytoplasmic fractions of the HepG2 cells. Analysis is based solely on the log ratios of the mean normalized intensity values from the hybridization of the reverse-transcribed amplified RNA samples to the miRNA microarrays. The Cluster and Treeview programs that are found on the GEPAS website [69] were used to compare 1)nuclear to cytoplasmic, 2)nuclear to total, and 3)cytoplasmic to total ratios, which are color-coded to represent the log ratios of the mean intensity values as indicated. The clustering was performed with complete linkage using the euclidean distance, and the unweighted pair group method with arithmetic averages.

The list of 156 miRNA probes that showed a significant difference between the nuclear and cytoplasmic fractions was reduced to 121 to eliminate some redundancies. For the 121 probes, the log-ratio data comparing the mean, normalized intensities for nuclear to cytoplasmic, nuclear to total, and cytoplasmic to total RNA fractions were subjected to cluster analysis (Fig. 5). Most of the pri-miRNAs formed two groups that both contained pri-miRNAs more concentrated in the nucleus. A much smaller group of pri-miRNAs hybridized less intensely with the nuclear samples when compared to the cytoplasm and/or the total RNA fractions, indicating a higher pri-miRNA concentration for these in the cytoplasm. One of the pri-miRNA groups that was higher in the nucleus had only 4 members, but they showed particularly high nuclear to cytoplasm ratios for their intensities. The only human miRNA pri-miRNA in this group was for let-7e, but the group also contained the probe for mouse miR-329. The other two members, were hybridized to the probes for mouse miR-106a and mouse miR-325. The corresponding human pri-miRNAs fell into the larger group with high nuclear to cytoplasmic ratios.

## Discussion

The primary purpose of these experiments was to explore the suitability of using nuclear RNA, as compared to total RNA, or its predominant component, cytoplasmic RNA, for studying global gene expression. Our interest in nuclear RNA was based on two practical considerations: first that nuclei could be directly purified at 4°C from cellular homogenates using fluorescence-activated sorting [24,25]; and second, that nuclei could be labeled through transgenic expression and targeting of Fluorescent Proteins within specific cell types [21,22]. Thus, combining high RNA integrity with the ability to isolate genetic material in a cell-type specific manner provided a unique approach for studying gene expression in select cell types. Our previous studies [22,25], which employed higher plants, served as the model for extending this approach to mammalian cells, with the aim of validating it and establishing its generality for multicellular eukaryotes. Evidently, global analyses of cytoplasmic RNA transcripts appear more likely to reflect the patterns of protein biosynthesis at any particular time, whereas analyses of nuclear transcripts appear more likely to track the process of transcription. It therefore was of interest to explore the global similarities and differences between the transcript populations in these two cellular locations, and for this purpose microarrays were employed. Two microarray platforms were available; in the first case, the oligonucleotide array elements represented annotated gene transcripts. In the second case, the array elements represented microRNAs. To simplify the biological system, we employed human HepG2 cells growing in culture, which represent a relatively homogeneous population of cells. Evaluation using a Bioanalyzer (Agilent, Santa Clara, CA) indicated that we were able to prepare RNA of excellent quality from the sorted HepG2 nuclei, and this RNA was sufficient in quantity for microarray target preparation using one round of amplification. Microarray hybridization was highly reproducible, yielding a list of 744 transcripts for the human genome platform and 156 transcripts for the miRNA platform, that were expressed at significantly different intensities in the nuclear and cytoplasmic RNA fractions, based on a criterion of a false discovery rate of less than 0.05.

Considering first the analysis of annotated, protein-coding gene transcripts, we were able to demonstrate that nuclear RNA hybridization patterns were very similar to those obtained using either total or cytoplasmic RNA. When transcripts of the nuclear and the cytoplasmic compartments were compared, only 3% of all transcripts were found at significantly different levels. Approximately-equal numbers of gene transcripts were enriched within and depleted from the nucleus (389 versus 354 transcripts, respectively). This observation demonstrates that an analysis of nuclear transcripts can be used as an accurate gauge of the general global pattern of transcript regulation for a cell, and it validates an important step of the proposed strategy of employing flow sorting for enrichment of the nuclei of specific cell types, followed by nuclear transcript profiling [41].

The observation that a small minority of the transcripts were significantly enriched in the nucleus or cytoplasm raises the question as to the biological purpose of this enrichment, and the related question as to how it might be achieved. The demonstration of differential expression within two cell fractions is evidence for the relative purity of the nuclear fractions, and evidence that the segregation of transcripts is an important function for the cell. The enrichment of some transcripts in the nucleus with respect to the cytoplasm may suggest that the rate of transcription for these genes is relatively high, and conversely, their rate of release to the cytosol low and/or their rate of degradation in the cytosol is high [42]. Enrichment of transcripts in the cytoplasm with respect to the nucleus could also imply that the stability of the transcripts is relatively high, and that their transcription rates are low.

Other explanations for the selective enrichment of transcripts within one subcellular compartment could include the physical association of the RNA with specific cellular structures. For example, some mRNAs, including those coding membrane proteins and glycoproteins, are associated by polyribosomal translation with the endoplasmic reticulum (ER) membranes. Transcripts for proteins destined for various other endomembrane locations are also expected to be associated with the ER. Since the ER has functional continuity with the outer nuclear membrane, this could explain the enrichment of membrane protein transcripts with the nuclei [43-45]. In that some of the proteins produced in the ER are specifically targeted to the nucleus, this would explain the enrichment in the nuclear fractions of some of the transcripts for nuclear proteins [44,46,47].

Clues to the purpose of the spatial segregation of transcripts within the cell may be found in the annotational analysis of the nucleus-enriched and cytoplasm-enriched transcripts. For example, the ontology categories that were overrepresented in the list of transcripts enriched in the nucleus included the cell cycle and the ubiquitin cycle. Both categories relate to rapid changes in the programming of the cell. Additionally, transitions of state associated with operation of the cell cycle require rapid changes in both the transcriptome and in the proteome, the latter being regulated by ubiquitination and protein degradation within proteasomes. Thus, the purpose of the spatial segregation of transcripts may be to regulate the activity of a functional pathway, by controlling which transcripts are expressed constitutively in the cytoplasm, and which ones are held in the nucleus away from the translational machinery.

**Figure 6:**
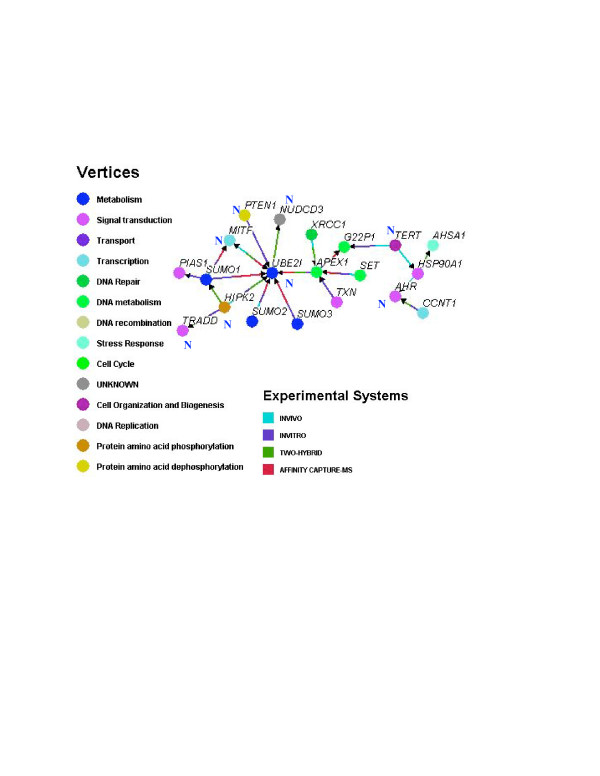
The lists of nucleus-enriched and cytoplasm-enriched transcripts were analyzed for potential interactions by the proteins represented by the transcripts. The analysis was performed with Osprey software, which employs The Biogrid [49], a database of protein-protein interactions based on in vitro, in vivo, yeast two-hybrid system, and affinity-capture mass spectrometry experimentation. The main grouping of interacting proteins that resulted is presented here. Those proteins that represented nucleus-enriched transcripts are marked with an 'N'. The proteins are labeled with the corresponding gene name, and the annotation information for the proteins is provided in Table 1. Each protein is color coded according to its annotation heading. The experimental system(s) employed to determine the protein-protein relationship is indicated by the color coding of the arrows.

The potential importance of regulation by separation of transcripts is illustrated in Fig. 6, which was created with the program Osprey, a protein-interaction visualization tool [48]. Osprey was used to consider possible interactive relationships between the proteins coded by the genes that are enriched in either the nucleus or the cytoplasm. The interactions identified by Osprey are documented from experiments *in vitro *and *in vivo*, by yeast two-hybrid studies, and by affinity-capture mass spectrometry, all integrated into a single database, The Biogrid [49]. The network created using Osprey (Figure 6) represents a small fraction of the 389 nucleus-enriched and 354 cytoplasm-enriched genes, but helps illustrate how the separation could be important to some regulatory pathways. The network linked together some of the protein products of nucleus-enriched transcripts through a central ubiquitin-conjugating enzyme, *UBE2I *(Table 1). Including cytoplasm-enriched transcripts in the analysis enlarged the network to include both nuclear-enriched and cytoplasm-enriched transcripts. The nuclear enriched transcripts represented in this linked pathway were related to apoptosis (*PTEN1*, *MITF*, *TRADD, AHR, TERT*), the cell cycle (*PTEN1*, *HIPK2, AHR*), and the stress response (*AHR*). The cytoplasm-enriched transcripts included three small ubiquitin modifiers (*SUMO1, SUMO2*, and *SUMO3*), as well as other proteins related to DNA repair (*APEX1, XRCC1*, and *G22P1*), the cell cycle (the small ubiquitin modifiers [50], and cyclin T1), and the stress response (*AHSA1*, and the 90 kDa heat shock protein, *HSP90AA1*). The network defined by Osprey suggests that transcripts that are expressly segregated within the cell, the cytoplasm-enriched transcripts and the cytoplasm-depleted transcripts, encode proteins that may interact in regulating the closely-interrelated functions of the cell cycle, DNA repair, the ubiquitin cycle, apoptosis, and the stress response. At least one transcript that was retained in the nucleus (*UBE2I*) occupied a central position in this network linking together several other components.

**Table 1:** Proteins in the Interaction Network

Name	Description	Go Component	Go Process
*AHR*	aryl hydrocarbon receptor	nucleus	cell cycle;response to stress;apoptosis
*AHSA1*	AHA1, activator of heat shock 90 kDa ATPase homolog 1 (yeast)	ER;cytoplasm	response to stress;protein folding
*APEX1*	APEX nuclease (multifunctional DNA repair enzyme) 1	nucleus;ER;ribosom	base-excision repair;DNA repair
*CCNT1*	cyclin T1	nucleus	cytokinesis;regulation of cell cycle
*G22P1*	X-ray repair in Chinese hamster cells 6	nucleus	double-strand break;DNA repair
*HIPK2*	homeodomain interacting protein kinase 2	nucleus;nuclear body;cytoplasm	apoptosis;reg. of cell cycle
*HSP90A1*	heat shock 90 kDa protein 1, alpha	cytosol	mitochondrial transport;protein refolding
*MITF*	microphthalmia-associated transcription factor	nucleus	regulation of transcription; melanocyte differentiation
*NUDCD3*	NudC domain containing 3	NONE	NONE
*PIAS1*	protein inhibitor of activated STAT, 1	nucleus	ubiquitin cycle;regulation of transcription
*PTEN1*	phosphatase and tensin homolog (mutated in cancers 1)	cytoplasm	negative reg. of cell cycle;induction of apoptosis
*SET*	SET translocation (myeloid leukemia-associated)	ER;perinuclear region;nucleus	nucleocytoplasm transport;DNA replic.;nucleosome assembly
*SUMO1*	SMT3 suppressor of mif two 3 homolog 1 (yeast)	nucleus	ubiquitin cycle;protein modification;protein sumoylation
*SUMO2*	SMT3 suppressor of mif two 3 homolog 2 (yeast)	nucleus	ubiquitin cycle;protein modification
*SUMO3*	SMT3 suppressor of mif two 3 homolog 3 (yeast)	kinetochore	ubiquitin cycle;protein modification
*TERT*	telomerase reverse transcriptase	chromosome, telomeric region;nucleus	RNA-dependent DNA replication;telomere maintenance
*TRADD*	TNFRSF1A-associated via death domain	NONE	induction of apoptosis
*TXN*	thioredoxin	NONE	electron transport;cell-cell signaling;cell motility;cell prolif.
*UBE2I*	ubiquitin-conjugating enzyme E2I (UBC9 homolog, yeast)	NONE	ubiquitin cycle;protein modification
*XRCC1*	X-ray repair in Chinese hamster cells 1	intracellular;nucleus	single strand break repair

A novel mechanism for the nuclear retention of transcripts has been previously proposed [51-53]. Prasanth et al. demonstrated that the transcript for *SLC7A2*, a mouse cationic amino acid transporter, was normally localized to the nucleus. After stress induction by treatment with α-amanitin, a large portion of the *SLC7A2 *transcripts were redistributed into the cytoplasm. Further, they showed that retention was related to adenosine to inosine edits found in the 3'UTR of the nuclear transcripts, and that cleavage of this edited portion was required for the translocation into the cytoplasm. The adenosine to inosine edits were the result of the activity of a well-documented enzyme, adenosine deaminase, which acts on double-stranded RNA, and was previously shown to cause the retention of viral RNA in the nucleus [53,54].

The proposed model for nuclear retention [51] could explain the enrichment of transcripts in the nuclear fraction with respect to the cytoplasm observed in our studies. Prasanth et al. [51] have proposed that the nuclear retention of the *SLC7A2 *transcript is a component of the response of the cell to stress, and that more generally, nuclear retention of transcripts could be an important stress response mechanism. In our experiments, we did not see a statistically significant overrepresentation of transcripts for the stress response in the nuclear-enriched transcripts, with an exception for the subcategory of stress-activated protein kinase signaling. Nonetheless, we found specific stress-related genes among our nuclear-enriched transcripts, including one for another cationic amino acid transporter important to the stress response, *SLC7A11 *[55]. We also found a significant presence of ubiquitin cycle components among the nuclear-enriched transcripts, the ubiquitin cycle being also important to the stress response [56,57]. Our data suggest a possible broader role for nuclear retention that includes other processes that require a rapid change in the cell program.

Conclusive information as to whether the nuclear retention model is in play will require complete analysis of the sequences of 3'UTRs of the transcripts enriched with respect to the cytoplasm. Adenosine to inosine edits can be detected by comparing multiple transcript sequences for the same gene, since the edited adenosines are represented as guanosines in cDNA sequences. In a search of all human transcripts available in Genbank, Levanon et al. [58] found 1,637 genes with variant transcripts that had adenosine to inosine edit sites. 92% of the edit sites were associated with ALU repeats, which were also associated with the hairpin structures described in the 3'UTR of SLC7A2 transcripts [51]. We compared our list of 389 nuclear-enriched transcripts to the list of transcripts identified by [59]. Of the 292 transcripts of our list having annotated gene names, which allowed cross-referencing between the two lists, 22 (7.5%) also appeared in the list of transcripts containing adenosine to inosine editing sites [59]. This supports the idea that some of the nuclear-enriched transcripts can be variants that are retained in the nucleus. The other transcripts on our list of nuclear-enriched transcripts also may have adenosine to inosine editing sites, but these sites remain to be identified.

The nuclear retention model would best explain much of the spatial segregation of transcripts implied by our data. Localization of mRNA may relate most directly to the regulation of some processes within the cell. Nuclear retention may hold transcripts aside, untranslated, until they are needed by the cell. Rapid release of the transcripts to the cytoplasm would allow fast expression of key proteins, for example, *UBE2I *in the protein interaction network described above. Without this key protein, important ubiquitin-mediated pathways may be inactive, until the transcript for *UBE2I *is released from the nucleus.

In terms of transcripts not destined for translation, such as ribosomal RNA (rRNA) and miRNA, we would expect that nuclear RNA would be enriched for primary or precursor forms of these transcripts. The primary transcript of rRNA contains both 18S and 28S rRNA sequences. Processing of the transcript takes place in the nucleoli, where the individual rRNA components are assembled into the ribosomes [60]. miRNA is processed in a more complex fashion, being produced first as a large, polyadenylated primary transcript (pri-miRNA), which is processed within the nucleus by the Drosha ribonuclease into an intermediate precursor form (pre-miRNA), which is transported to the cytoplasm and then cleaved by the Dicer RNAse to the final, active miRNA [14,32]. Very little of the full-length transcript ever reaches the cytoplasm [15,16].

Our data obtained using miRNA-specific arrays confirmed that the levels of pri-mRNAs were elevated within nuclear extracts when compared to the cytoplasm [15,16]. The RNA purification and amplification protocols that we employed limited our studies to the polyadenylated pri-miRNA, and excluded consideration of mature miRNA. Our data (Figure 5) indicated that the majority (72%) of the pri-miRNA transcripts were enriched in the nucleus.

A relatively small number of pri-miRNAs were slightly enriched in the cytoplasm with respect to the nucleus. One would not expect pri-miRNAs ever to be higher in the cytoplasmic or total fractions. Either this small group of transcripts consists of exceptions to the general rule, or a number of artifacts may have created this result. One such artifact could result from leakage from damaged nuclei, which could be compounded by the much longer preparation time for the nuclear samples (approximately 1–1.5 hr vs. less than 15 min for cytoplasmic samples, which were prepared from a separate flask). The longer preparation time for the nuclear samples, though maintained at 4°C, may also allow Drosha to selectively reduce the nuclear signal. Contamination of the cytoplasmic RNA with nuclear RNA is not likely to be an important factor in itself. Nuclear RNA typically makes up approximately 10–15% of the total RNA, and so even if half of the nuclear RNA contaminated the cytoplasmic RNA, the maximal contamination would be 8%, a proportion too small to permit the cytoplasm to be more enriched in a putatively contaminating transcript than the nucleus itself. A more trivial artifact may result from cross-hybridization with mRNA, which certainly could be the case for the probes for non-human miRNAs that make up a disproportionate fraction (67%) of this group. It is also possible that some miRNAs are not completely processed in the nucleus before passage to the cytoplasm. Some miRNAs are transcribed within the introns of protein coding transcripts, and some are found within the exons or introns of non-translated mRNA-like transcripts. These transcripts could possibly be processed outside of the nucleus [61].

## Conclusion

The nucleus serves a central role in the programming of a cell, and so it is not unusual that multiple processes are reflected in the enrichment of transcripts either within the nuclear compartment or away from the nucleus in the cytoplasm. Clearly the current cell program is well represented by the new transcripts produced in the nucleus, and these new transcripts may first appear as precursors or partially processed transcripts, as in the case of pri-miRNA. Some transcripts in the nucleus may be untranslated variants that are retained until they may be rapidly processed and transported to the cytoplasm when needed by the cell. Other transcripts may be associated with our nuclear fractions because they are enriched in a part of the ER or other membrane structure connected to the nucleus. Some transcripts may be routed directly to the cytosol for immediate translation. The localization of transcripts within the cell provides clues to the regulation of the RNA species or the proteins that they may code. Much more study is needed before we have a more comprehensive view of how the movement and segregation of transcripts function in cellular programming. Specifically we plan to sequence the transcripts segregated within the cell to determine if they have adenosine to inosine modifications. We will also examine further the localization of pri-miRNAs within the cell, to determine whether some of them have unprocessed or partially processed forms that reach the cytoplasm.

## Methods

### Cell culture

HepG2 human hepatoma cells (American Type Culture Collection, Manassas, VA) were grown in Dulbecco's Modification of Eagle's Medium without L-glutamine, supplemented with 4.5 gm/L glucose, 110 mM sodium pyruvate, 15 mM HEPES, 10% fetal calf serum, penicillin, streptomycin, and Glutamax-I (1X, Invitrogen, Carlsbad, CA). Cells were cultured to approximately 80% confluence in 75 cm^2 ^flasks prior to harvest. Four biological replicate samples were prepared on different days. Each biological replicate employed 5 flasks plated with cells at the same time. For each day, separate flasks were processed simultaneously to produce nuclear, cytoplasmic, and total RNA samples.

### Preparation of nuclear RNA

Three 75 cm^2 ^flasks of HepG2 cells were placed on ice and incubated for 5 min with 10 ml of ice-cold HMS buffer (8% Sucrose, 25 mM KCl, 5 mM MgCl_2_, 20 mM Tris-HCl, pH 7.4). The buffer was withdrawn and replaced with 2 ml HMS, and the cells were scraped from the flask surface with a plastic cell scraper. The cells were immediately homogenized on ice in a Dounce homogenizer, with 10 strokes of a loose-fitting pestle, followed by 30 strokes with a tight-fitting pestle. The homogenate was filtered through a 40 μm nylon mesh screen, and the volume adjusted to 3 ml with HMS. DAPI (4',6-diamidino-2-phenylindole) was added to a final concentration of 0.67 μg/ml and incubated for 10 min at 4°C. The homogenate was layered on top of a 2-step gradient consisting of 5 ml layers of 12.5% and 35% iodixanol. The layers were prepared by mixing the 60% stock solution of iodixanol (Sigma, St.Louis, MO) 5:1 with 0.12 M Tris-HCl, pH 7.4, 0.15 M KCl, 30 mM MgCl_2 _to make buffer HMI. Buffers HMI and HMS were mixed in appropriate proportions to constitute the gradient layers. The homogenate and gradient were centrifuged for 25 min at 4500 × g at 4°C, and the lower interface, containing the nuclei, was removed. This sample was very gently diluted to 4 ml with HMS. The nuclei were further purified using a MoFlo flow cytometer/cell sorter (Dako North America, Inc., Carpinteria, CA), equipped with a Coherent Enterprise II laser providing 50 mW at 365 nm and 200 mW at 488 nm. The filter configuration routed DAPI fluorescence to a 450/65 bandpass filter with 90° light scatter (side scatter) being detected using a 95/5 dichroic and a 480/10 barrier filter. Samples were sorted at a rate of 500–1000 nuclei/s. Data were visualized as biparametric histograms of log DAPI vs. log side scatter which were triggered on side scatter. A sort window was selected to minimize contaminants, especially from smaller cell particles. We examined preparations of nuclei by light and epifluorescence microscopy, and found that they contained largely nuclei and a small amount of free membranes. The sorted nuclei were collected directly into RLT buffer (Qiagen, Valencia CA), at a ratio of 0.4 nuclei to 2 ml RLT. Nuclear samples were either frozen at -80°C or RNA was extracted immediately, according to the Qiagen RNeasy Minikit protocol. A DNase treatment step was included in the preparation protocol according to the Qiagen instructions. Cells and nuclei were maintained at 4°C to reduce the possibility of transcription in the nuclei or degradation of the RNA. Additionally, the DAPI used to stain the nuclei was well above the IC50 for inhibition of initiation of transcription by RNA polymerase II [62].

### Preparation of cytoplasmic RNA

Cytoplasmic RNA was prepared according to the Qiagen (Valencia, CA) RNeasy Minikit protocol. A 75 cm^2 ^flask of HepG2 cells (separate from those used for the nuclear preparation) was placed on ice, the growth medium was removed and 1.75 ml of RLN buffer (50 mM Tris-HCL pH 7.4, 0.14 M NaCl, 1.5 mM MgCl_2_, 0.5% IGEPAL 630 detergent, with 40 U/ul RNasin (Promega Biosciences, Inc., Madison, WI) was added. After incubating on ice for 5 min, the cells were scraped from the flask, and the resulting homogenate was centrifuged for 3 min at 300 × g at 4°C. The supernatant was combined with 6 ml Qiagen RLT (lysis) buffer and RNA was prepared according to the Qiagen instructions or frozen at -80°C. A DNase treatment step was included in the preparation protocol according to the Qiagen instructions.

### Preparation of total RNA

The growth medium was removed from a 75 cm^2 ^flask of HepG2 cells, and 8 ml of Qiagen (Valencia, CA) RLT buffer was added. The cells were scraped free from the flask with a plastic cell scraper and the RNA was prepared according to the Qiagen instructions or frozen at -80°C. A DNase treatment step was included in the preparation protocol according to the Qiagen instructions.

### RNA amplification

RNA sample quality was assessed with an Agilent 2100 Bioanalyzer (Agilent Technologies, Inc. Santa Clara, CA). Sample concentration was determined with the Nanodrop ND-1000 Spectrophotometer (Nanodrop, Inc, Wilmington, DE). RNA samples were amplified using the Ambion MessageAmp kit [30]. Aminoallyl-UTP was included in the RNA transcription step according to the Ambion instructions. The amplified RNA (aRNA) was labeled with Amersham CyDye Post-Labeling Reactive Dye Packs (GE Healthcare Products, Piscataway, NJ) by vacuum drying 4 μg of the aRNA, and resuspending it in 4.5 μl 0.2 M NaHCO_3_, and then adding 4.5 μl Cy3 or Cy5 dye. Dye was prepared by adding 22 μl DMSO to the contents of a freshly-opened tube of dye. The aRNA was incubated in the dark at room temperature for 2 hours, and the reaction was terminated by the addition of 4.5 μl of 4 M hydroxylamine. After incubation in the dark at room temperature for 15 min, the reaction was diluted with 100 μl of nuclease-free water, and then purified according to the Qiagen (Valencia, CA) RNeasy Mini kit protocol. The purified and labeled aRNA was assessed and quantified with the Nanodrop ND-1000 Spectrophotometer.

### Hybridization of human genomic arrays

A total of 12 arrays were hybridized for each array type, using an experimental design which emphasized direct comparisons within a slide between the cytosolic fraction and the nuclear and total fractions, randomizing assignments with respect to day [see Additional file 2]. Samples from the same cell fractions for the same day were labeled with different dyes on different slides to control for dye effects. The human genomic oligo microarrays, printed with the Operon Human Genome Oligo Set V2.0, were purchased from the Gladstone Institute of the University of California San Francisco. The microarrays were rehydrated and snap-dried 3 times to expand the DNA spots. After each rehydration step they were irradiated with 120 mJ uv in a Stratalinker (Stratagene, Inc., LaJolla, CA). The arrays were washed for 10 min with agitation in 0.1% SDS, then rinsed with nuclease-free water, and dried rapidly under a nitrogen stream. The hybridization mixture consisted of 100 pMoles of Cy3 or Cy5 dye conjugated to aRNA, in 100 μl of 2X SSC, 0.08% SDS, and 6% Liquid Block (GE Healthcare Products, Piscataway, NJ), and was applied under a glass 24 × 60 mm LifterSlip (Erie Scientific, Portsmouth, NH) to the array surface. Hybridization was performed at 55°C for 7 hr in a humidified chamber. The arrays were washed successively for 5 min with 2X SSC with 0.5% SDS at 55°C, 0.5X SSC at room temperature, and twice with 0.05X SSC. The arrays were rapidly dried under a nitrogen stream, and scanned with a Genepix 4200AL scanner (Molecular Devices, Sunnyvale, CA) using 635 nm and 532 nm lasers. The Genepix 6.0 software was used for quantitation of the scanned images.

### Reverse-transcription of aRNA for miRNA microarrays

aRNA (3 μg) was incubated at 42°C for 2 hr with 1.5 μl Powerscript (Clontech, Mountain View, CA), 25 μg/ml random hexamers, 0.25 mM aminoallyl-dUTP, 0.625 mM dCTP, 0.625 mM dATP, 0.625 mM dGTP, 0.375 mM dTTP, and 30 units RNAsin (Promega Biosciences, Inc., Madison, WI) in the Powerscript buffer. The RNA was hydrolyzed by incubation with 90 mM NaOH and 90 mM EDTA at 65°C for 15 min. The pH of the cDNA was neutralized with 1 M TRIS-HCl pH 7.0 (19 μl). The cDNA was purified by addition of 26 μl 0.1 M NaAcetate, followed by the Qiagen (Valencia, CA) Qiaquick protocol. The cDNA was coupled to Cy3 or Cy5 dyes by using the protocol described above for aRNA. Cleanup of the cDNA was by repetition of the Qiaquick protocol.

### Hybridization of the miRNA microarrays

miRMax miRNA microarrays were purchased from the W.M. Keck Center for Collaborative Neuroscience (The State University of New Jersey, Rutgers, NJ). The slides were washed and incubated with all of the cDNA from a single reverse transcription reaction (1–1.5 μg total) as described for the human genomic arrays above, except that 24 × 30 mm LifterSlips with 80 μl volume was used, and the hybridization temperature was 42°C. Scanning and quantitation was as given above.

### Data and Statistical Analyses

Similar protocols were used to analyze separately the data from the human genomic and miRNA arrays. To account for spot saturation and multiple spots with the same intensity, quantile normalization was performed [63] using cumulative percentages rather than overall ranks. Following normalization, data from saturated spots were deleted from the data set. Data manipulations and analyses were completed in SAS 9.0 (SAS Institute, Cary, North Carolina USA). For each gene, a mixed model ANOVA was performed [64], modeling cell fraction and dye as fixed effects, and slide, day, and spot nested within slide as random effects. The FDR was computed using the qvalue routine [65,66] implemented in R [67]. Any effect in the ANOVA model for a given gene with FDR < 0.05 was considered for further analysis. Significance of pair-wise contrasts among least-squares means following ANOVA was determined for genes with FDR < 0.05. All p-values for contrasts were used to compute the FDR. Contrasts with FDRs < 0.05 were defined as significant for the purposes of this study.

### Data annotation

Annotation and further analysis were achieved with the aid of the Clone/Gene ID converter [68] and Cluster and Treeview [69] of the Gene Expression Pattern Analysis Software Suite v3 [70], and the GO Toolbox [28]. Gene ontology (GO) annotation and the analysis of over- and under-representation of genes in different GO categories was performed with GoToolbox [27]. Additionally the software, Osprey v. 1.2 [48,49], was employed to examine the relationships of proteins coded by the transcripts that were enriched in the nucleus (with respect to the cytoplasm), and the cytoplasm (with respect to the nucleus).

## Abbreviations

miRNA (microRNA); pri-miRNA (primary microRNA); pre-miRNA (hairpin form of microRNA or microRNA processed by Drosha, but not Dicer); ER (endoplasmic reticulum); ANOVA (analysis of variance).

## Competing interests

The author(s) declares that there are no competing interests.

## Authors' contributions

RAB-All nuclear preparations, RNA extractions, amplifications, microarray work, annotational analysis, and main writing of the manuscript.

GML- Developed methods for and performed flow cytometry.

CV- Created hybridization design and performed statistical analysis of microarray data.

RML- Helped develop approach for experiments and helped in final analysis of annotated data.

DWG- Developed the approach for experiments, helped develop nuclear preparation method and flow cytometry methods, and helped in final analysis of annotated data.

All contributed to the writing of the manuscript, and all read and approved the final manuscript.

## Supplementary Material

Additional file 1 Statistical results for all genes represented on the human genomic microarray. The normalized means of median intensities for all the scans, plus information relating to the statistical analysis, including the q values (adjusted p values), and the significant trends (higher or lower) for each comparison. Click here for file

Additional file 2 Hybridization plan for both human genomic microarrays and microRNA arrays. This describes specifically on which array each sample is hybridized. Click here for file

## References

[B1] Hughes TR, Marton MJ, Jones AR, Roberts CJ, Stoughton R, Armour CD, Bennett HA, Coffey E, Dai H, He YD, Kidd MJ, King AM, Meyer MR, Slade D, Lum PY, Stepaniants SB, Shoemaker DD, Gachotte D, Chakraburtty K, Simon J, Bard M, Friend SH (2000). Functional discovery via a compendium of expression profiles. Cell.

[B2] Moore MJ (2002). Nuclear RNA turnover. Cell.

[B3] Orphanides G, Reinberg D (2002). A unified theory of gene expression. Cell.

[B4] Kloc M, Zearfoss NR, Etkin LD (2002). Mechanisms of subcellular mRNA localization. Cell.

[B5] Teixeira D, Sheth U, Valencia-Sanchez MA, Brengues M, Parker R (2005). Processing bodies require RNA for assembly and contain nontranslating mRNAs. RNA.

[B6] Bhattacharyya SN, Habermacher R, Martine U, Closs EI, Filipowicz W (2006). Relief of microRNA-mediated translational repression in human cells subjected to stress. Cell.

[B7] Rana TM (2007). Illuminating the silence: understanding the structure and function of small RNAs. Nat Rev Mol Cell Biol.

[B8] Cheng J, Kapranov P, Drenkow J, Dike S, Brubaker S, Patel S, Long J, Stern D, Tammana H, Helt G, Sementchenko V, Piccolboni A, Bekiranov S, Bailey DK, Ganesh M, Ghosh S, Bell I, Gerhard DS, Gingeras TR (2005). Transcriptional maps of 10 human chromosomes at 5-nucleotide resolution. Science.

[B9] Carninci P (2006). Tagging mammalian transcription complexity. Trends Genet.

[B10] Carninci P, Sandelin A, Lenhard B, Katayama S, Shimokawa K, Ponjavic J, Semple CA, Taylor MS, Engstrom PG, Frith MC, Forrest AR, Alkema WB, Tan SL, Plessy C, Kodzius R, Ravasi T, Kasukawa T, Fukuda S, Kanamori-Katayama M, Kitazume Y, Kawaji H, Kai C, Nakamura M, Konno H, Nakano K, Mottagui-Tabar S, Arner P, Chesi A, Gustincich S, Persichetti F (2006). Genome-wide analysis of mammalian promoter architecture and evolution. Nat Genet.

[B11] Gingeras TR (2006). The multitasking genome. Nat Genet.

[B12] Carninci P, Kasukawa T, Katayama S, Gough J, Frith MC, Maeda N, Oyama R, Ravasi T, Lenhard B, Wells C, Kodzius R, Shimokawa K, Bajic VB, Brenner SE, Batalov S, Forrest AR, Zavolan M, Davis MJ, Wilming LG, Aidinis V, Allen JE, Ambesi-Impiombato A, Apweiler R, Aturaliya RN, Bailey TL, Bansal M, Baxter L, Beisel KW, Bersano T, Bono H (2005). The transcriptional landscape of the mammalian genome. Science.

[B13] Katayama S, Tomaru Y, Kasukawa T, Waki K, Nakanishi M, Nakamura M, Nishida H, Yap CC, Suzuki M, Kawai J, Suzuki H, Carninci P, Hayashizaki Y, Wells C, Frith M, Ravasi T, Pang KC, Hallinan J, Mattick J, Hume DA, Lipovich L, Batalov S, Engstrom PG, Mizuno Y, Faghihi MA, Sandelin A, Chalk AM, Mottagui-Tabar S, Liang Z, Lenhard B (2005). Antisense transcription in the mammalian transcriptome. Science.

[B14] Lee Y, Jeon K, Lee JT, Kim S, Kim VN (2002). MicroRNA maturation: stepwise processing and subcellular localization. EMBO J.

[B15] Lee Y, Ahn C, Han J, Choi H, Kim J, Yim J, Lee J, Provost P, Radmark O, Kim S, Kim VN (2003). The nuclear RNase III Drosha initiates microRNA processing. Nature.

[B16] Cai X, Hagedorn CH, Cullen BR (2004). Human microRNAs are processed from capped, polyadenylated transcripts that can also function as mRNAs. RNA.

[B17] Denli AM, Hannon GJ (2003). RNAi: an ever-growing puzzle. Trends Biochem Sci.

[B18] Brandt S, Kehr J, Walz C, Imlau A, Willmitzer L, Fisahn J (1999). Technical Advance: A rapid method for detection of plant gene transcripts from single epidermal, mesophyll and companion cells of intact leaves. Plant J.

[B19] Grebenok RJ, Pierson E, Lambert GM, Gong FC, Afonso CL, Haldeman-Cahill R, Carrington JC, Galbraith DW (1997). Green-fluorescent protein fusions for efficient characterization of nuclear targeting. Plant J.

[B20] Grebenok RJ, Galbraith DW, Penna DD (1997). Characterization of Zea mays endosperm C-24 sterol methyltransferase: one of two types of sterol methyltransferase in higher plants. Plant Mol Biol.

[B21] Chytilova E, Macas J, Sliwinska E, Rafelski SM, Lambert GM, Galbraith DW (2000). Nuclear dynamics in Arabidopsis thaliana. Mol Biol Cell.

[B22] Zhang C, Gong FC, Lambert GM, Galbraith DW (2005). Cell type-specific characterization of nuclear DNA contents within complex tissues and organs. Plant Methods.

[B23] Pozner-Moulis S, Pappas DJ, Rimm DL (2006). Met, the hepatocyte growth factor receptor, localizes to the nucleus in cells at low density. Cancer Res.

[B24] Galbraith DW, Harkins KR, Maddox JM, Ayres NM, Sharma DP, Firoozabady E (1983). Rapid Flow Cytometric Analysis of the Cell Cycle in Intact Plant Tissues. Science.

[B25] Galbraith D, Lambert G, Macas J, Dolezel D, Robinson JP, Darzynkiewicz Z, Dean PN, Dressler LG, Rabinovitch PS, Stewart CV, Tanke HJ, Wheeless LL (1997). Analysis of nuclear DNA content and ploidy in higher plants. Current Protocols in Cytometry.

[B26] Goff LA, Yang M, Bowers J, Getts RC, Padgett RW, Hart RP (2005). Rational probe optimization and enhanced detection strategy for microRNAs using microarrays. RNA Biol.

[B27] GOToolBox. http://crfb.univ-mrs.fr/GOToolBox/.

[B28] Martin D, Brun C, Remy E, Mouren P, Thieffry D, Jacq B (2004). GOToolBox: functional analysis of gene datasets based on Gene Ontology. Genome Biol.

[B29] Benjamini Y, Drai D, Elmer G, Kafkafi N, Golani I (2001). Controlling the false discovery rate in behavior genetics research. Behav Brain Res.

[B30] Eberwine J (1996). Amplification of mRNA populations using aRNA generated from immobilized oligo(dT)-T7 primed cDNA. Biotechniques.

[B31] Gu J, He T, Pei Y, Li F, Wang X, Zhang J, Zhang X, Li Y (2006). Primary transcripts and expressions of mammal intergenic microRNAs detected by mapping ESTs to their flanking sequences. Mamm Genome.

[B32] Lee Y, Kim M, Han J, Yeom KH, Lee S, Baek SH, Kim VN (2004). MicroRNA genes are transcribed by RNA polymerase II. EMBO J.

[B33] Fu H, Tie Y, Xu C, Zhang Z, Zhu J, Shi Y, Jiang H, Sun Z, Zheng X (2005). Identification of human fetal liver miRNAs by a novel method. FEBS Lett.

[B34] Babak T, Zhang W, Morris Q, Blencowe BJ, Hughes TR (2004). Probing microRNAs with microarrays: tissue specificity and functional inference. RNA.

[B35] Chang J, Nicolas E, Marks D, Sander C, Lerro A, Buendia MA, Xu C, Mason WS, Moloshok T, Bort R, Zaret KS, Taylor JM (2004). miR-122, a mammalian liver-specific microRNA, is processed from hcr mRNA and may downregulate the high affinity cationic amino acid transporter CAT-1. RNA Biol.

[B36] Li SC, Pan CY, Lin WC (2006). Bioinformatic discovery of microRNA precursors from human ESTs and introns. BMC Genomics.

[B37] Tran T, Havlak P, Miller J (2006). MicroRNA enrichment among short 'ultraconserved' sequences in insects. Nucleic Acids Res.

[B38] miRBase::Sequences. http://microrna.sanger.ac.uk/sequences/.

[B39] Griffiths-Jones S, Grocock RJ, van Dongen S, Bateman A, Enright AJ (2006). miRBase: microRNA sequences, targets and gene nomenclature. Nucleic Acids Res.

[B40] Weber M (2005). New human and mouse microRNA genes found by homology search. FEBS J.

[B41] Galbraith D (2003). Global analysis of cell type-specific gene expression. Comp Funct Genomics.

[B42] Yugi K, Nakayama Y, Kojima S, Kitayama T, Tomita M (2005). A microarray data-based semi-kinetic method for predicting quantitative dynamics of genetic networks. BMC Bioinformatics.

[B43] Nicchitta CV (2002). A platform for compartmentalized protein synthesis: protein translation and translocation in the ER. Curr Opin Cell Biol.

[B44] Imreh G, Maksel D, de Monvel JB, Branden L, Hallberg E (2003). ER retention may play a role in sorting of the nuclear pore membrane protein POM121. Exp Cell Res.

[B45] Ellenberg J, Siggia ED, Moreira JE, Smith CL, Presley JF, Worman HJ, Lippincott-Schwartz J (1997). Nuclear membrane dynamics and reassembly in living cells: targeting of an inner nuclear membrane protein in interphase and mitosis. J Cell Biol.

[B46] Saksena S, Shao Y, Braunagel SC, Summers MD, Johnson AE (2004). Cotranslational integration and initial sorting at the endoplasmic reticulum translocon of proteins destined for the inner nuclear membrane. Proc Natl Acad Sci USA.

[B47] Deng M, Hochstrasser M (2006). Spatially regulated ubiquitin ligation by an ER/nuclear membrane ligase. Nature.

[B48] Breitkreutz BJ, Stark C, Tyers M (2003). Osprey: a network visualization system. Genome Biol.

[B49] The Biogrid. http://www.thebiogrid.org.

[B50] Gutierrez GJ, Ronai Z (2006). Ubiquitin and SUMO systems in the regulation of mitotic checkpoints. Trends Biochem Sci.

[B51] Prasanth KV, Prasanth SG, Xuan Z, Hearn S, Freier SM, Bennett CF, Zhang MQ, Spector DL (2005). Regulating gene expression through RNA nuclear retention. Cell.

[B52] Zhang Z, Carmichael G (2001). The Fate of dsRNA in the Nucleus: A p54nrb-Containing Complex Mediates the Nuclear Retention of Promiscuously A-to-I Edited RNAs. Cell.

[B53] Kumar M, Carmichael GG (1997). Nuclear antisense RNA induces extensive adenosine modifications and nuclear retention of target transcripts. Proc Natl Acad Sci USA.

[B54] Bass BL (2002). RNA editing by adenosine deaminases that act on RNA. Annu Rev Biochem.

[B55] Lewerenz J, Klein M, Methner A (2006). Cooperative action of glutamate transporters and cystine/glutamate antiporter system Xc- protects from oxidative glutamate toxicity. J Neurochem.

[B56] Brooks CL, Gu W (2003). Ubiquitination, phosphorylation and acetylation: the molecular basis for p53 regulation. Curr Opin Cell Biol.

[B57] Hurley JH, Lee S, Prag G (2006). Ubiquitin-binding domains. Biochem J.

[B58] Levanon EY, Hallegger M, Kinar Y, Shemesh R, Djinovic-Carugo K, Rechavi G, Jantsch MF, Eisenberg E (2005). Evolutionarily conserved human targets of adenosine to inosine RNA editing. Nucleic Acids Res.

[B59] Levanon EY, Eisenberg E, Yelin R, Nemzer S, Hallegger M, Shemesh R, Fligelman ZY, Shoshan A, Pollock SR, Sztybel D, Olshansky M, Rechavi G, Jantsch MF (2004). Systematic identification of abundant A-to-I editing sites in the human transcriptome. Nat Biotechnol.

[B60] Granneman S, Baserga SJ (2004). Ribosome biogenesis: of knobs and RNA processing. Exp Cell Res.

[B61] Rodriguez MS, Dargemont C, Stutz F (2004). Nuclear export of RNA. Biol Cell.

[B62] Chiang SY, Welch J, Rauscher FJ, Beerman TA (1994). Effects of minor groove binding drugs on the interaction of TATA box binding protein and TFIIA with DNA. Biochemistry.

[B63] Bolstad BM, Irizarry RA, Astrand M, Speed TP (2003). A comparison of normalization methods for high density oligonucleotide array data based on variance and bias. Bioinformatics.

[B64] Wolfinger RD, Gibson G, Wolfinger ED, Bennett L, Hamadeh H, Bushel P, Afshari C, Paules RS (2001). Assessing gene significance from cDNA microarray expression data via mixed models. J of Computational Biology.

[B65] Storey JD (2002). A direct approach to false discovery rates. J Roy Stat Soc Ser B (Stat Method).

[B66] Storey JD, Tibshirani R (2003). Statistical significance for genomewide studies. Proc Natl Acad Sci USA.

[B67] The R Project for Statistical Computing. http://www.r-project.org.

[B68] ID converter. http://idconverter.bioinfo.cipf.es.

[B69] GEPAS – GEPAS:Tools. http://gepas.bioinfo.cipf.es/cgi-bin/tools.

[B70] Vaquerizas JM, Conde L, Yankilevich P, Cabezon A, Minguez P, Diaz-Uriarte R, Al-Shahrour F, Herrero J, Dopazo J (2005). GEPAS, an experiment-oriented pipeline for the analysis of microarray gene expression data. Nucleic Acids Res.

